# Survival of Long-Lived Plasma Cells (LLPC): Piecing Together the Puzzle

**DOI:** 10.3389/fimmu.2019.00965

**Published:** 2019-05-03

**Authors:** Shivana M. Lightman, Adam Utley, Kelvin P. Lee

**Affiliations:** Department of Immunology, Roswell Park Comprehensive Cancer Center, Buffalo, NY, United States

**Keywords:** long-lived plasma cells (LLPC), plasma cell survival, plasma cell niche, plasma cell function, humoral responses

## Abstract

Durable humoral immunity is dependent upon the generation of antigen-specific antibody titers, produced by non-proliferating bone marrow resident long-lived plasma cells (LLPC). Longevity is the hallmark of LLPC, but why and how they survive and function for years after antigen exposure is only beginning to be understood. LLPC are not intrinsically long-lived; they require continuous signals from the LLPC niche to survive. Signals unique to LLPC survival (vs. PC survival in general) most notably include those that upregulate the anti-apoptotic factor Mcl-1 and activation of the CD28 receptor expressed on LLPC. Other potential factors include expression of BCMA, upregulation of the transcription factor ZBTB20, and upregulation of the enzyme ENPP1. Metabolic fitness is another key component of LLPC longevity, facilitating the diversion of glucose to generate pyruvate during times of stress to facilitate long term survival. A third major component of LLPC survival is the microenvironment/LLPC niche itself. Cellular partners such as stromal cells, dendritic cells, and T regulatory cells establish a niche for LLPC and drive survival signaling by expressing ligands such as CD80/CD86 for CD28 and producing soluble and stromal factors that contribute to LLPC longevity. These findings have led to the current paradigm wherein both intrinsic and extrinsic mechanisms are required for the survival of LLPC. Here we outline this diverse network of signals and highlight the mechanisms thought to regulate and promote the survival of LLPC. Understanding this network of signals has direct implications in increasing our basic understanding of plasma cell biology, but also in vaccine and therapeutic drug development to address the pathologies that can arise from this subset.

## Introduction

Plasma cells (PC) represent an essential arm in humoral immunity as the main line of defense against infection and re-infection. As the primary producers of circulating immunoglobulin (Ig), these cells provide vital durable and protective immunity against a multitude of pathogens. Longitudinal analysis of antigen-specific antibody titers from vaccinated humans demonstrates that the predicted half-life of the measles titer is 3,014 years (1). This is a testament to the long-lived protection that PC can provide. Unlike other immune cell subsets such as T cells or B cells, the complexity of the varying PC subsets is only beginning to be understood. Plasma cell generation occurs primarily upon T cell-dependent differentiation of B lymphocytes to PC in germinal center reactions (2, 3). The current paradigm proposes that two general types of PC develop from these interactions: short-lived plasma cells (SLPC) and long-lived plasma cells (LLPC) (4, 5). The LLPC subset characteristically thought to traffic to and reside in the bone marrow (BM), is the subset that provides long-term and sustained antibody production that is maintained for decades to the lifetime of an individual (6–8). The germinal center reactions, through somatic hypermutation and class-switch recombination, allow for the selection of high-affinity antibody producing PC, which is proposed to be the major precursors of LLPC (9, 10). However, there is relatively little understanding of the driving force behind why these LLPC can become long lived.

## Specialized Niches for LLPC

There has been considerable research into the biology of PC as a whole, from how they are generated and the key transcriptional programs involved, to their ability to traffic to various organs (11–15). Further studies have elucidated the cellular and molecular components of various organ-specific niches occupied by PC (16–18). However, there is relatively little understanding of what distinguishes the ability of LLPC to survive in contrast to SLPC. LLPC are not intrinsically long-lived, as their survival is critically dependent on the ability to access and use a fixed number of specific pro-survival niches in the BM, secondary lymphoid organs, mucosa, and sites of inflammation (5, 7, 19–24). The BM is traditionally thought to be the primary organ of LLPC residence. It provides a dynamic infrastructure amenable to the formation of a complex microenvironment and allows for the generation of cell-type specific niches more easily than other less plastic organs (5, 6).

More recent work has illuminated the fact that LLPC do not only reside within the BM. Of human PC, about 80% are located in gut-associated lymphoid tissue (GALT) and produce primarily IgA (25). This allows for tolerance to the commensal bacteria in our gut, while also providing protection against unfavorable microbial and dietary antigens. It was originally thought that continual activation of B cells within the mucosa supplied the pool of IgA-producing PC in the gut (26, 27). However, new studies have highlighted that PC in the gut can also persist for long periods of time. Antibodies specific to *Escherichia coli* were detected 112 days after exposure (28). Examination of intestinal biopsies kept in culture contained non-proliferating IgA-producing PC for >4 weeks (29). Another study showed that 9 months post-immunization with both an IgA-inducing antigen (cholera toxin) and a T-dependent antigen (Ova), antigen-specific PC could be detected in the Lamina Propria (LP) but also within the BM (21). This suggests that survival niches present in the gut could be similar to those in the BM and that mucosal PC can utilize these niches in the same way as BM PC, as well as contributing to the BM LLPC pool (21).

It is traditionally thought that most BM LLPC secrete IgG antibodies, and those that reside in mucosal sites such as the gut produce IgA antibodies, however, about 40% of BM PC also produce IgA (30). It has also been reported that there are long-lived low-affinity IgM producing PC within the BM that appear to occupy different niches (20). Furthermore, allergic sensitization with ovalbumin generates IgG, IgA, and IgE secreting PC that can be found in the BM for an extended period (31). Therefore, evidence suggests that the longevity of a PC is not primarily driven by its antibody isotype—but rather by the nature of the pathogen, the characteristics of initial B cell activation elicited by the pathogen, and the niche the resultant PC homes to. This broad framework suggests that the primary role of SLPC is in protection against frequent (and less severe) endemic infections that is sustained by recurrent and antigen-dependent B cell reactivation; whereas LLPC provide sustained protective immunity against infrequent (but more severe) epidemic infections in an antigen-independent fashion. It has also been proposed that early B cell/plasmablast activation signals determine whether PC enter and respond to survival signals in the PC niche (32–34). Because the space available to LLPC is finite, LLPC longevity requires both access to the survival niche and the ability to respond to the niche's unique pro-survival signals.

## LLPC and Memory B Cells Are Not One and the Same

It was originally proposed that sustained antibody responses resulted from constant replenishment of a SLPC pool by continuous memory B cell re-stimulation (35). However, observations that BM transplantation caused non-allergic individuals to acquire allergies through transfer of allergen-specific IgE production lent credence to the idea that these PC were long-lived, due to the absence of antigenic re-stimulation in these patients (36, 37). Further studies have shown *in vivo* that some PC subsets are indeed long-lived and that absence of antigen plus depletion of memory B cells through radiation did not abrogate the ability of this LLPC subset to continue to produce high-affinity antigen-specific antibodies (38, 39). Other studies have shown that prolonged therapeutic depletion of the total B cell pool did not affect antigen-specific BM PC numbers or antibody titers in vaccinated murine models (40), nor did it affect antibody titers against childhood vaccines in humans (41). In the human studies, vaccine-specific antibody titers were maintained following anti-CD20 monoclonal antibody treatment (which targets B cells but not PC), despite clear depletion of the memory B cell pool (41). Sustained B cell aplasia caused by CD19 CAR-T cells (which also target B cells but not PC) in adult and pediatric acute lymphocytic leukemia patients also had no effect on serum vaccine antigen-specific antibody titers nor PC numbers in either the BM or ileum and colon (42). A study examining PC dynamics from biopsies of transplanted duodenum found that 1-year post-transplantation, CD38^+^ PC from the donor could still be identified. Further characterization identified CD19^−^ and CD19^+^ PC present in these biopsies, where the CD19^−^ PC subset represented a stable population with a potentially long lifespan (24). Lastly, characterization of subsets of human PC demonstrated that CD19^−^ PC in the BM were predominantly IgG secreting with a mature phenotype and remained settled in the BM after systemic vaccination (43). This demonstrates that LLPC are indeed distinct from other B cell subsets, however the key factors that distinguish these cells are only recently beginning to be characterized.

## A Unique Transcriptional Profile

During B cell to PC differentiation, many genes are downregulated including those involved in antigen presentation and BCR function. Concurrently, there is upregulation of the PC lineage defining transcription factor *PRDM1* (BLIMP-1) and genes involved in protein translation and the unfolded protein response (*XBP-1*) (11, 44). An avenue to distinguish this LLPC subset is through their transcriptional profile. A recent study of human PC has established a unique profile that distinguishes between early PC, circulating blood PC, and long-lived BM-resident CD138^+^ PC (14). The signature that distinguishes LLPC contains a significant number of genes that are downregulated and only a handful of genes that are upregulated. This latter group includes the anti-apoptotic genes *Mcl-1* and *ZNF667*, ER stress-associated genes including *EROILB* and *MANF*, the cation transport ATPase *ATP12A*, and *TFBS* and *SRF* that play roles in retention of hematopoietic progenitor cells in the BM. Many of these genes are associated with the central function of PC to produce significant amounts of protein in the form of immunoglobulin—although the fact that this function is not unique to LLPC suggests that there may be additional unappreciated biology of these genes in LLPC.

Identification of a transcriptional profile that is unique to LLPC faces a significant experimental obstacle—namely the ability to identify a pure population of LLPC to analyze. Anatomic location has traditionally been the way that SLPC and LLPC were distinguished from each other, due to the lack of distinct phenotypic markers distinguishing these subsets. However, as noted above, recent sophisticated phenotypic profiling has made it increasingly clear that LLPC are not the only PC present within the BM. Recent work looking at PC precursor populations has identified that several waves of trafficking can occur post-antigen exposure and PC differentiation—resulting in other PC aside from LLPC being in the BM (34). Furthermore, surface markers that were traditionally used to identify LLPC have been recently shown to variably stain different PC subsets—for example, analysis of PC in the BM compartment have shown that there is varying expression of surface markers such as CD38 and CD19 (43, 45). These phenotypic differences are clearly associated with functional differences, such as whether these PC are producing high-affinity or low-affinity antibodies (46).

Because of the recent increase in PC subset heterogeneity, analysis of the total BM PC population compared to PC from other compartments may not pinpoint the specific factors and interactions responsible for reinforcing and sustaining the transcriptional signature necessary for the LLPC subset. Thus, one approach in defining the key transcriptional networks unique to LLPC, is to first identify the upstream receptors and signal transduction pathways that uniquely support LLPC survival and longevity.

## Intrinsic Signals for LLPC Survival and Function

### BCMA

We now know that there are intrinsic signals uniquely utilized by LLPC that regulate their long-term survival and function (Table 1). It is well-established that factors such as A Proliferation Inducing Ligand (APRIL), Interleukin-6 (IL-6), B cell Activating Factor (BAFF), CD44 and CXCL12 can support survival of both SLPC and LLPC in various organs (4, 12, 29, 47–49). One of the receptors for both BAFF and APRIL is B cell maturation antigen (BCMA), which is highly upregulated on PC (50). Genetic knockout of *Tnfrsf17* (which encodes BCMA) resulted in a significant loss of antibody secreting cells (ASCs) in the BM compared to wild-type (Wt) controls, 6–8 weeks post-immunization. However, germinal center responses and early antigen-specific serum IgM and IgG titers were normal, indicating loss of BCMA primarily affected the LLPC subset (50). Additional studies have demonstrated that murine memory B cell survival is not dependent on BCMA, and BCMA is only induced in human B cells committed to PC differentiation (47, 51). This suggests that reliance on BCMA for survival is specific to the PC compartment. Furthermore, studies in multiple myeloma (MM), a BM-resident Ig-class switched LLPC neoplasm, showed that BCMA significantly promoted MM survival and proliferation (52). Therapeutically targeting BCMA in MM has shown high selectivity against the MM and PC populations (53), supporting a PC-restricted role for the receptor. Activation of BCMA by BAFF increases expression of the anti-apoptotic molecule Mcl-1, pointing to the mechanistic basis by which the BAFF/BCMA axis promotes long-term survival (50). An increase in the gene expression for Mcl-1 in BM PC was observed in Wt mice compared to BM PC from BCMA^−/−^ mice, where both Mcl-1 mRNA and protein expression were significantly lower in the knockouts (54). Furthermore, loss of Mcl-1 causes a significant decrease in antigen-specific titers 21 days post immunization, consistent with an effect on the LLPC subset (54). As with BCMA, Mcl-1 is also a critical factor for MM survival (55, 56), which is consistent with the broader concept that much of myeloma biology is in fact the biology of normal LLPC (57).

**Table 1:** Summary of intrinsic factors supporting LLPC survival.

**Intrinsic Factors of LLPC survival**	
BCMA	Upregulation of anti-apoptotic genes
STAT3	Responds to IL-6, IL-10, and IL-21 signaling in PC, initiating downstream survival signaling associated with these cytokines
Aiolos	Promotes high-affinity antibody producing PC.
CD93	Possible connection with BLIMP-1—regulating mature LLPC phenotype and production of high-affinity antibodies
CD28	Signaling through the Vav/Grb2 motif can induce NFκB signaling and BLIMP-1 expression. This receptor can engage ligands CD80/CD86 to promote back signaling through DC and upregulation of IL-6
Autophagy (Atg5)	Recycling mechanism—supplying metabolic substrates and elimination of misfolded protein
Metabolic Profile	LLPC uptake glucose for antibody glycosylation. They also utilize glycolysis and mitochondrial pyruvate import under non-optimal conditions
ENPP1	Enzyme that is a glucose homeostasis-regulator of glucose and the metabolic pathway in LLPC

### STAT3

The importance of IL-6 in PC function and survival has been extensively studied (49, 58). IL-10 and IL-21 have also been implicated in human PC maturation and survival (59–61). IL-21 is predominantly produced by T follicular helper (Tfh) cells (62), which are involved in many phases of the humoral immune response from B cell activation to maturation and differentiation of B memory cells into PC (63). A study looking at human tonsil, splenic, lymph node, and BM PC indicated that BM PC had very low expression of IL-21R and that IL-21 only enhanced Ig secretion from secondary lymphoid PC but not BM PC (64). More recent work, however, has postulated that Tfh cells influence blood Ag-induced PC, which can contain precursors of the LLPC subset (65). Whether this effect is directly through IL-21 or the effect of IL-21 on B cell subsets and upregulation of receptors like IL-6R is uncertain. How these individual cytokines promote BM PC survival and function is still unclear; however, they all initiate STAT3 signaling (66–68). Activation of STAT3 plays an important role in the ability of PC to respond to these various cytokine signals (68). Impairment of this pathway inhibits cytokine-dependent increase in viability and IgG secretion. Furthermore, STAT3 activation appears to be a prerequisite for the ability of PC to respond to APRIL and BAFF. This suggests a critical role for STAT3 as a key downstream signaling node transducing stimuli in LLPC that reinforces their survival and longevity (68).

### Aiolos

Other factors restricted to the lymphoid lineage have been studied for their possible role in supporting the LLPC subset. Aiolos, part of the Ikaros gene family of nuclear regulators that function in modulating chromatin structure, is one of these factors (69). Absence of this gene does not affect B cell development or the early SLPC response but does affect long-term PC survival (70). Immunization normally causes a first wave of low-affinity antibodies produced primarily by SLPC, and then a second wave of higher affinity antibodies from BM LLPC (10). Upon deletion of Aiolos, there is a significant decrease in the production of high-affinity antibodies in the BM 120 days after primary immunization with no effect on the low-affinity antibody response (71). Additional studies show that the deletion of this gene does not cause a homing defect. This suggests that Aiolos upregulation is required for high-affinity antigen-specific LLPC, unlike other factors like BLIMP-1, which are required for generation of all PC. The exact mechanism remains to be understood, but the ability of Aiolos to promote high-affinity antibodies is important for enhanced protective immunity long term.

### CD93

Another starting point for identifying specific receptors involved in LLPC survival and longevity are those receptors that are highly upregulated on BM PC compared to mature B cells. Greenlee et al. observed that CD93, which has been largely characterized for its role in intercellular adhesion and clearance of apoptotic cells, is not present on mature B cells but is highly upregulated during PC differentiation (72). Upon immunization with either T-independent (TI) or T-dependent (TD) antigens, CD93 is further upregulated in BM PC, and the CD93^+^CD138^+^ PC have increased expression of BLIMP-1, XBP-1, IRF4, and produce higher-affinity antibodies—suggesting that this receptor identifies the LLPC subset (73). Consistent with this, loss of CD93 diminished the persistence of long-term antibody titers post immunization (73). Loss of Blimp-1 decreases CD93 expression only in PC, as CD93 is still present at low levels in Blimp-1-deficient immature B cells. This connection to Blimp-1, which is most highly expressed in LLPC, led the authors to hypothesize that CD93 is necessary to promote the mature phenotype of LLPC and production of high-affinity antibodies. This is further corroborated by a recent study looking at measles titers of vaccinated school children in which the most highly expressed genes in high responder groups were CD93, IL-6, and CXCL12- implicating CD93 involvement in the generation of long-lived antibody responses in humans (74).

### CD28

Upon activation, B cells downregulate the B cell lineage-defining transcription factor Pax5 (75). Pax5 was found to directly inhibit *CD28* gene expression in B cells, and the downregulation of Pax5 leads to upregulation of CD28 as an immediate/early event in B → PC differentiation. In this study loss of CD28 impaired short term induction of antigen-specific antibody titers, although long-lived antibody responses were not assessed. Previous studies in CD28 global knockouts and signaling-deficient CD28 receptor knock-in mice had demonstrated significant defects in antibody responses (76–79), but this was attributed to defects in T cell help. Subsequent work from our lab has shown that CD28 has an essential PC-intrinsic role in the survival of LLPC and the maintenance of durable antigen-specific IgG antibody titers after vaccination (80). Compared to Wt mice, unvaccinated CD28^−/−^ mice have comparable numbers of splenic PC but have significantly fewer BM PC. *In vitro* studies demonstrated that CD28 activation alone (without a “signal 1” that is necessary for T cells) enhances the survival of purified BM PC, but not splenic PC, in response to serum-starvation-induced death. *In vivo*, the loss of CD28 expression specifically in the B cell lineage (B cells do not express CD28, only PC) resulted in the inability to sustain antigen-specific antibody titers in these murine models (80). Work done by Njau et al. also looked at the effect of CD28 on PC. Initial studies with global CD28^−/−^ mice showed a significant increase in IgM antigen-specific responses compared to Wt mice, but found a significantly lower antigen-specific IgG response compared to Wt controls 60 days post immunization, which they attributed to a defect in T-cell help (81). However, in contrast to Rozanski et al. specific loss of CD28 in the B cell lineage resulted in a significant increase in antigen-specific antibody titers compared to Wt PC. The basis of the differences between these two studies is unclear, although the immunization strategies (both the antigen and in particular the adjuvants used) were different. Regardless both studies establish that CD28 is present on PC and has a role in affecting antibody responses.

Studies of signaling pathways emanating from the CD28 receptor in LLPC demonstrated that loss of downstream Vav/Grb2 mediated PLCγ1 signaling caused a loss of BM LLPC, while loss of downstream PI3K/AKT signaling had no effect (82). Interestingly, although murine SLPC also express CD28, direct activation of the receptor does not induce downstream activation of PLCγ1 and NFκB—indicating a signaling basis for why SLPC are not responsive to CD28 and suggesting that CD28 has different activation thresholds in SLPC vs. LLPC. Further downstream, CD28 activation augments BLIMP-1 expression, a master regulator of PC identity and antibody production (82, 83). Consistent with the role CD28 plays in normal LLPC, we have also found that activation of CD28 confers a major pro-survival/chemotherapy resistance signal in MM (84, 85).

Activation of CD28 on MM directly transduces a pro-survival signal, and also signals back to dendritic cells (DC) through the CD28 ligands CD80 and CD86 expressed on these DC (84–86). This back signaling through CD80/CD86 upregulates both IL-6 and the immunosuppressive enzyme indoleamine 2,3-dioxygenase (IDO) expression/activity in the DC to promote further MM survival. Thus, CD28 both directly delivers a pro-survival signal to the myeloma cell and modulates the DC in the niche to further support MM survival. Conversely, *in vivo* blockade of the CD28-CD80/CD86 interaction with CTLA4-Ig (which is FDA approved for the treatment of rheumatoid arthritis) significantly enhanced the sensitivity to chemotherapy in a murine model of MM (84). Given the significant shared biology between LLPC and MM, this bidirectional signaling axis between CD28 and CD80/CD86 is now being investigated as previously unrecognized pro-survival factors for LLPC.

### Autophagy

Because LLPC are continually generating and secreting high levels of Ig protein, they require unique metabolic pathways to deal with the protein load and ER stress. Induction of the Unfolded Protein Response (UPR) has been clearly identified as essential for B cells to differentiate to PC (87). A second mechanism implicated in the adaptation to this stress is autophagy. Autophagy's primary function is to sustain cellular metabolism when nutritional starvation occurs. In yeast, to counterbalance the stress caused by ER expansion, autophagic trimming is employed to remove excess ER (88). Within the immune compartment, mature T cells rely on autophagy-mediated turnover of mitochondria (mitophagy) to control intracellular production of reactive oxygen species (89, 90). Autophagy also appears to be critical for LLPC survival. Because antibody-producing PC are highly biosynthetic, it would be predicted that autophagy plays key roles in both processing/eliminating/recycling misfolded immunoglobulins as well as supporting the fuel and metabolic substrate requirements of the PC—in particular since the BM microenvironment may face highly variable nutrient availability due to changing levels of hematopoiesis during stress (91, 92). Analysis of the secretome from mesenchymal stromal cells (known to be part of the BM niche) identified two major survival factors of BM PC, fibronectin (FN-1) and the YWHAZ protein, which were shown to downregulate mTORC1 signaling in BM PC (17). Autophagy induction normally occurs after inhibition of mTOR (target of rapamycin), an evolutionarily-conserved protein kinase. mTOR is a key regulator in cell growth and response to the nutrient status and stress signals in a cell- and is known to negatively regulate autophagy (93). Thus, these factors in the niche could further influence the ability of LLPC to upregulate autophagy as a survival mechanism. Other studies have demonstrated that in mice knocked out for *Atg5*, which plays a central role in initiating autophagy, there is a significant defect in high-affinity LLPC post-immunization with a TD antigen (94). While all PC are generating Ig at high levels, it is possible that LLPC are more dependent on autophagy over their lifespan for both elimination of misfolded protein as well as a recycling mechanism to supply metabolic substrates, to reinforce the metabolic fitness of these cells during periods of nutrient deprivation.

### Metabolism

Recent studies have begun to connect the metabolic energy production pathways used by LLPC to those used by memory T cells (which need to rapidly respond to activation to become highly biosynthetic and proliferate). CD28 co-stimulation in naïve T cells switches their fuel utilization to fatty acids and mitochondrial oxidation to sustain their metabolic demands, and this has been shown to be required for the generation of memory T cells (95, 96). CD28 co-stimulation also induces changes in mitochondria in memory T cells—elongated mitochondria with increased cristae structure are associated with enhanced (spare) respiratory capacity compared with naive T cells. Similarly, work on the metabolic phenotype of LLPC by Lam et al. has interestingly shown that both mouse and human LLPC have a greater maximal respiratory capacity than SLPC, and although they have increased ability to take up glucose (via Glut1 upregulation), they constitutively use long chain fatty acids as their primary fuel source—and glucose appears to be primarily used for antibody glycosylation (97, 98). However, under possibly non-optimal conditions they are also capable of using glycolysis and mitochondrial pyruvate import. The importance of this “alternative” metabolism program was demonstrated by the finding that loss of the ability to import pyruvate into the mitochondria significantly decreased LLPC frequency and abrogated sustained long-lived antibody response 22 weeks post vaccination (97). Similar work looking at IgA-secreting PC in the LP exhibited higher expression of glycolysis-related metabolites than naïve B cells in Peyer's Patches (PP) (99). It is proposed that the switch from IgM to IgA requires a metabolic shift and preferential use of the glycolytic pathway. This group also observed that splenic SLPC did not have very many glycolytic metabolites- indicating that both the BM and gut are unique microenvironments that could contribute to this shift in the metabolic phenotype of LLPC.

### ENPP1

Further investigation into the potentially unique metabolic characteristics of LLPC has led to the discovery of other previously unrecognized pathways. The transmembrane glycoprotein ectonucleotide pyrophosphatase/phosphodiesterase 1 (ENPP1) is highly expressed on PC, but its function has only recently been demonstrated. This enzyme was previously characterized for its role in bone formation, glucose homeostasis and downregulation of the insulin signaling pathway (100). ENPP1^−/−^ mice were also found to have a significant reduction of BM LLPC 12 weeks post immunization with TD dependent antigen and *C. chabaudi* infection, but no effect was observed on splenic PC (101). ENPP1^−/−^ PC take up less glucose than their Wt counterparts and exhibit an impairment in glycolysis, suggesting a role for ENPP1 as a regulator of glucose and the metabolic pathway found in LLPC (101).

### Parallels Between Mice and Men

The relevance of some of the above factors in supporting LLPC survival were recently supported by characterization of human BM PC. These studies elegantly demonstrated the heterogeneity of BM PC in mice is also evident in humans, of which only a fraction appear to be the bonafide long-lived subset. This study defined 4 subsets of PC found in the BM: CD19^+^CD38^hi^CD138^−^ (subset A), CD19^+^CD38^hi^CD138^+^ (subset B), CD19^−^CD38^hi^CD138^−^ (subset C), and CD19^−^CD38^hi^CD138^+^ (subset D) (45). Subset D did not proliferate in comparison to the other three subsets and was CD20 negative, HLA-DR negative but CD28 positive (45). This subset had the highest number of IgG specific antibodies against tetanus, measles, mumps, and influenza. Interestingly, this subset also showed a distinctive RNA transcriptome signature for enhanced autophagy. This finding suggests that those components uncovered in murine studies are relevant to human PC and are fundamental to LLPC function and survival.

## Extrinsic Signals and Contribution of the Niche

### General PC Partners: Stromal Cells, Megakaryocytes, and Basophils

**Figure 1:**
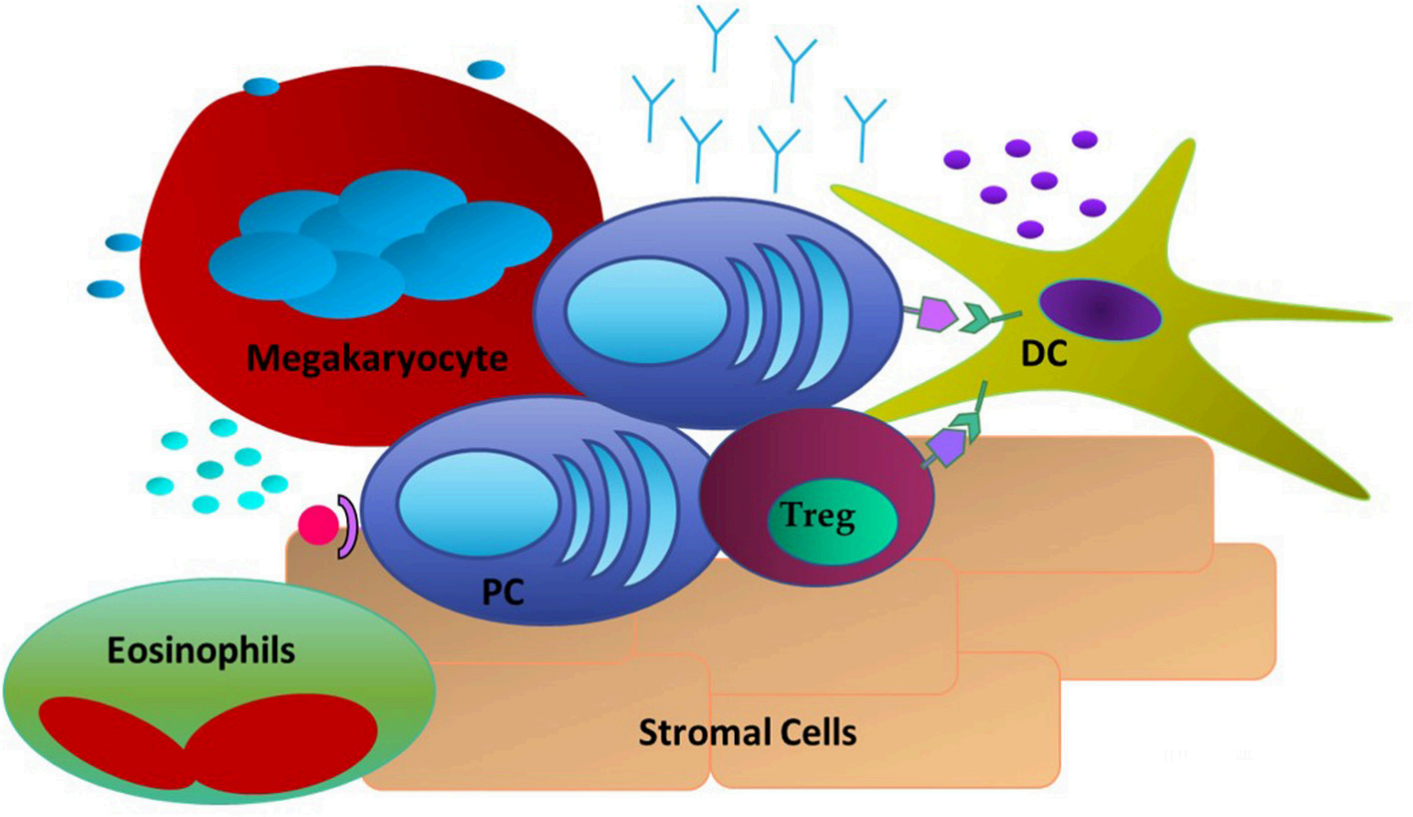
Extrinsic Factors in the LLPC niche. PC are associating with DC through CD28: CD80/CD86 interactions and with Tregs. Stromal cells expressing CXCL12 act as a homing signal to CXCR4^+^ PC. Megakaryocytes and Eosinophils are also present and have been shown to produce soluble factors such as APRIL and BAFF to promote PC survival.

The PC-intrinsic signaling and downstream responses detailed above do not occur in a vacuum. LLPC are critically dependent upon signals they receive from the niche, as LLPC cultured alone *in vitro* rapidly die (102). Studies of the LLPC BM niche have identified several different cell types that can provide survival signals to the PC (Figure 1). The first cell type to be identified were reticular and mesenchymal stromal cells within the BM. They have high expression of CXCL12, the ligand for CXCR4 expressed on PC, which is responsible for their trafficking into the BM (18, 103). However, even though initial studies demonstrated these stromal cells supported PC survival *in vitro*, this survival was not sustained—and *in vivo*, they did not appear to have an essential role in supporting LLPC survival (48, 104). Furthermore, the PC-survival factors like APRIL, BAFF, and IL-6 are not secreted by these cells. Yet, it has recently been shown that BM mesenchymal stromal cells secrete fibronectin (FN-1) and other proteins (YWHAZ) that can sustain LLPC survival *in vitro*, in combination with hypoxia and factors like APRIL (17). FN-1 binds integrins, collagen and CD138 (highly expressed on LLPC), and is proposed to play an important role in tethering PC to the BM extracellular matrix. Studies in MM have also shown that integrin-mediated binding to fibronectin facilitates resistance to chemotherapy-mediated killing (105).

Other studies have identified megakaryocytes as part of the LLPC niche. Megakaryocytes can produce APRIL and IL-6, and mice that do not have the ability to form megakaryocytes have a reduced number of BM PC when compared to Wt mice (106). Furthermore, upon immunization with ovalbumin (Ova), it was observed that Ova-specific PC were in direct contact with megakaryocytes in the BM. However, functional studies of PC post-immunization in the absence of megakaryocytes did not show any effect in the late stage of antigen-specific antibody responses. Basophils are another cell type reported to support PC survival. In *in vitro* cultures of basophils and BM PC, the presence of basophils supported the survival of these PC and markedly increased the Ig production of these cells (107). However, functional studies of the effect of basophils on antigen-specific BM PC responses were only assessed *in vitro*, therefore it is still uncharacterized how these cells might affect BM LLPC in the *in vivo* setting. It was determined that the survival effects seen by basophils are most likely due to their ability to secrete IL-4 and IL-6. Thus, megakaryocytes, and basophils may play an important role in establishing the LLPC niche and possibly LLPC access, however, it remains unclear how they contribute to long term survival and maintenance of LLPC.

### Eosinophils

Another cell subset thought to provide critical survival cytokines to PC, like IL-6, and APRIL, are eosinophils. It was reported that eosinophils co-localize with PC in both the BM and gut associated lymphoid tissue (GALT) and that eosinophil-deficient mouse have significantly fewer PC at steady state and post-exposure to antigen (108, 109). For IgA-secreting PC, eosinophils are necessary for promoting IgA production and maintaining immune homeostasis in the GALT (110). In a study of PC in the lamina propria one-third of the PC were found to be localized next to eosinophils (21). With respect to the BM niche, it was found that lack of eosinophils caused LLPC in the BM to undergo apoptosis. Upon reconstitution of eosinophils, the number of PC was transiently increased, suggesting a role for these cells in homing of PC to the BM as well as maintaining the PC population once there (108). Once activated an eosinophil can promote B cell differentiation into IgM secreting PC that is independent of the induction of germinal centers and generation of high-affinity IgG secreting PC (111). However, more recent studies have questioned whether eosinophils are a necessary component for LLPC survival and function, at least in murine models this has not been conclusive (112). Therefore, the current literature suggests that eosinophils may play a critical role in the generation and trafficking of PC, for both mucosal sites and the BM, but their contribution to the survival, maturity, and long term function of LLPC remains unsettled.

### Dendritic Cells

Parallel studies in the niche have focused on cellular components that express the ligands that can engage receptors on LLPC that support survival and function. Our work has demonstrated a key role for CD80, and CD86, the natural ligands for CD28. An absence of these ligands in global knockout mice significantly diminished BM LLPC numbers and durable antigen-specific titers (80). The cells that have the highest expression of CD80 and CD86 are myeloid (conventional) DC. Other myeloid cells (monocytes, eosinophils) can also have significant expression. Our lab and others have demonstrated co-localization of DC with PC occurs within the BM. Additionally this supports LLPC survival *in vitro* (in a contact and CD28-dependent fashion) and *in vivo* (80, 113). This interaction has further significance as DC can produce significant amounts of the pro-PC survival cytokine IL-6, and that CD80/CD86 activation is one pathway that induces DC IL-6 production (86). As noted above, DC also significantly enhances the survival of MM in a CD28-CD80/CD86 dependent manner. This suggests a bidirectional signaling axis in the niche to support LLPC both directly and indirectly through modulation of cellular partners to produce essential survival factors.

### T Regulatory Cells

Another significant partner recently identified within the LLPC BM niche are T regulatory (Tregs) cells in close physical association with CD11c^+^ DC. High number of Tregs have been shown to co-localize with PC in the BM *in vivo*, and this is necessary to homeostatically support BM PC populations (113). Ablation of Tregs during infection caused a significant reduction in the number of BM PC. The phenotype of these BM Tregs was consistent with an activated subset with increased suppressive capabilities, and most closely matched the profile observed in injured muscle. Interestingly, unlike splenic Treg cells that have little CTLA-4 expression, these BM Tregs express CTLA-4—which is a member of the CD28 superfamily that also binds CD80 and CD86. In the absence of Treg expression of CTLA-4, there was a 3-fold increase in the number of BM PC. The authors proposed that CTLA-4^+^ Tregs contribute to the maintenance of an immune-privileged niche for LLPC in part by buffering this niche against systemic inflammatory changes, as well as maintaining LLPC homeostasis by limiting the PC pool (113). However, what role CTLA-4 itself is playing within the PC niche is unclear. It is possible that through expression on Tregs it acts in a homeostatic way to limit the amount of PC able to traffic into and reside within the BM. However, it could also be binding to the CD80/CD86 receptors on niche DC, causing upregulation of cytokines like IL-6 to help support PC survival. Alternatively, Treg CTLA-4 may compete for and limit LLPC CD28 binding to these ligands on the DC, as part of homeostatic regulation of the LLPC population. It is interesting to speculate that this cellular triumvirate of LLPC, DC, and Tregs is modulated by dynamic molecular interactions between CD28, CD80/CD86, and CTLA-4, facilitating a stable niche that critically supports LLPC survival and continued function.

## Concluding Remarks

This review has identified some of the components that form the networks that allow for survival and maintenance of LLPC. Long-term generation of protective antibodies by this subset is crucial for durable protective immunity. Understanding these key molecular and cellular components that support LLPC survival and longevity gives us tools for utilization of new vaccination strategies as well as therapeutic strategies against diseases that arise from this cell, such as MM and autoimmune syndromes.

It has become clear that there are distinct subsets of PC defined by anatomic location, phenotype, function, and longevity (Table 2). Evidence suggests that PC making high affinity neutralizing Ig (which would be particularly important in rapidly lethal pathogens that characteristically cause epidemics) are key members of the LLPC subset and are generated upon germinal center reactions and then traffic to the BM; where they have unique characteristics that allow them to unlock and occupy/utilize the LLPC niche. It is now clear that this is a significant oversimplification given the heterogeneity of the BM PC populations and niches present in other organs like the gut. Nonetheless, a growing body of evidence demonstrates that the “traditional” LLPC that produce lifelong protective antibody titers, requires both PC intrinsic and niche components to be long-lived. These interactions can then lead to downstream PC-intrinsic signaling that buttresses the ability of the LLPC subset to survive over decades in their microenvironment; which in the BM is hypoxic and crowded with rapidly proliferating cells (especially reactive BM responding to infection) competing for space and nutrients, and in mucosal sites where there is constant exposure to both helpful and harmful microbes. Thus, the key aspects that underlie the longevity of LLPC likely reinforce the mechanisms that allow for effective adaptation to these stresses over a lifetime.

**Table 2:** Importance of intrinsic factors in B cell and PC subsets.

**Intrinsic factors**	**Cell type**	**Expression**	**Differentiation**	**Survival**	**References**
BCMA	*Mature B cell*	Yes	Not necessary	Not necessary	(114)
	*SLPC*	Yes	Not necessary	No	(50)
	*LLPC*	Yes	Not necessary	Yes	(50)
	*Mucosal LLPC*	Yes	Unknown	Yes	(115)
STAT3	*Mature B cell*	Yes	Yes	Yes	(116)
	*SLPC*	Yes	Yes	Yes	(68)
	*LLPC*	Yes	Yes	Yes	(68)
	*Mucosal LLPC*	Unknown	Unknown	Unknown	
Aiolos	*Mature B cell*	Yes	Yes	Necessary for homeostasis	(70)
	*SLPC*	Yes	Not necesssary	Not necessary	(71)
	*LLPC*	Yes	Yes	Yes	(71)
	*Mucosal LLPC*	Unknown	Unknown	Unknown	
CD93	*Mature B cell*	No	Not necesssary	Not necessary	(117)
	*SLPC*	Yes	Yes	Not necessary	(73)
	*LLPC*	Yes	Yes	Yes	(73)
	*Mucosal LLPC*	Unknown	Unknown	Unknown	
CD28	*Mature B cell*	No	No	No	(75)
	*SLPC*	Yes	No	No	(80)
	*LLPC*	Yes	No	Yes	(80)
	*Mucosal LLPC*	Yes	No	No	(118)
Autophagy	*Mature B cell*	Yes	Not necesssary	Yes	(119)
	*SLPC*	Yes	Yes	Yes	(120, 121)
	*LLPC*	Yes	Yes	Yes	(94)
	*Mucosal LLPC*	Unknown	Unknown	Unknown	
Metabolic shift: glycolysis and mitochondrial alterations	*Mature B cell*	Yes	Yes	Yes	(122)
	*SLPC*	No	No	No	(97, 99)
	*LLPC*	Yes	Yes	Yes	(97)
	*Mucosal LLPC*	Yes	Yes	Yes	(99)
ENPP1	*Mature B cell*	Yes (terminally differentiated GC B cells)	No	Unknown	(101, 123)
	*SLPC*	Yes	No	No	(101)
	*LLPC*	Yes	No	Yes	(101)
	*Mucosal LLPC*	Unknown	Unknown	Unknown	

## Author Contributions

SL primary author and drafted the bulk of the review, substantial contributions to conception and design. AU provided revisions to content of manuscript. KL contributed to conception and design, provided critical revisions and final approval, and provided funding.

### Conflict of Interest Statement

The authors declare that the research was conducted in the absence of any commercial or financial relationships that could be construed as a potential conflict of interest.
